# Hyperhemolysis Syndrome in a Patient With Sickle Cell Disease and Acute Chest Syndrome

**DOI:** 10.7759/cureus.13017

**Published:** 2021-01-30

**Authors:** Karthik Shankar, Deep Shah, Deanna L Huffman, Chelsea Peterson, Rama Bhagavatula

**Affiliations:** 1 Internal Medicine, Allegheny Health Network, Pittsburgh, USA; 2 Hematology/Oncology, Allegheny Health Network, Pittsburgh, USA

**Keywords:** nucleated red blood cells, hyperhemolysis syndrome, sickle cell anemia, acute chest syndrome, packed red blood cell transfusion, hemoglobin, hemoglobinopathies, reticulocyte count, sickling, methylprednisolone

## Abstract

Sickle cell anemia patients often present to the hospital with acute vaso-occlusive pain crisis. Symptoms can include, but are not limited to, chest pain, abdominal pain, and musculoskeletal pain. These symptoms are brought about due to the pathology of the disease. Abnormal hemoglobin S causes red blood cells to band together, otherwise known as "sickling." These patients also often present with very low hemoglobin levels on initial evaluation. In most cases, packed red blood cell transfusions are needed in order to replenish these patient's functional hemoglobin supply. Unfortunately, transfusing sickle cell patients can lead to an unwanted consequence, that of hyperhemolysis syndrome, in which blood transfusions prompt further hemolysis of the already sickled red blood cells. When this complication arises, caution must be exercised in deciding the next steps of treatment.

## Introduction

Sickle cell anemia (SCA) is a genetic disorder characterized by homozygous hemoglobin S [1]. Chronic hemolytic anemia and vaso-occlusive crisis from red blood cells (RBC) sickling are pervasive. Many patients require frequent blood transfusions. However, transfusion of blood in sickle cell anemia patients is not always beneficial. In the case of hyperhemolysis syndrome, transfusions can actually worsen hemolysis and subsequently cause life-threatening anemia [1].

We report a case of sickle cell disease and acute chest syndrome, complicated by hyperhemolysis syndrome.

A 34-year-old female, with history of sickle cell disease, presented with chest pain, shoulder pain, and vague abdominal pain. Her hemoglobin on admission was 6.4. Initial chest X-ray and CT chest were unremarkable. She was treated for vaso-occlusive pain crisis but on day 2 of her hospital course, patient’s respiratory status declined. She required intubation and a repeat chest X-ray showed new onset bilateral infiltrates. Thus a diagnosis of acute chest syndrome was made. She underwent two exchange transfusions requiring 12 units of packed red blood cells (PRBC). Despite significant improvement in her respiratory status, persistent elevation of lactate dehydrogenase (LDH) and bilirubin along with undetectable haptoglobin, increasing nucleated RBC count, severe reticulocytopenia raised the concern for hyperhemolysis syndrome. Transfusions were held and methylprednisolone 40 mg BID was started.

Over the course of day 7 to 11, the patient’s laboratory parameters improved and she was discharged in stable condition.

When giving blood transfusions to sickle cell patients in acute vaso-occlusive crisis or acute chest syndrome, hyperhemolysis syndrome should be a concern when combination of persistently high LDH and bilirubin along with undetectable haptoglobin, severe reticulocytopenia and increasing nucleated RBCs is seen. If the possibility is not accounted for when transfusing sickle cell patients, life-threatening anemia can ensue [1].

## Case presentation

Our patient is a 34-year-old female with past medical history of sickle cell disease who presented to the hospital with sharp severe chest pain and right shoulder pain of acute onset, starting that same morning. She had been experiencing on and off subjective fevers with associated nausea and fatigue for the preceding week leading up to admission. She felt short of breath when walking short distances with vague bilateral hip pain. Her urine was dark colored, but she denied hematuria or dysuria. Her last pain crisis from sickle cell disease was reported to be two years ago. Her home pain medication regimen was Tylenol 500 mg every six hours as needed, Ibuprofen 200 mg every six hours as needed, Tramadol 50 mg every six hours as needed, and Morphine 15 mg every four hours as needed. Her sickle cell disease was well under control and her last hematologist visit was nine months prior to admission.

**Figure 1:**
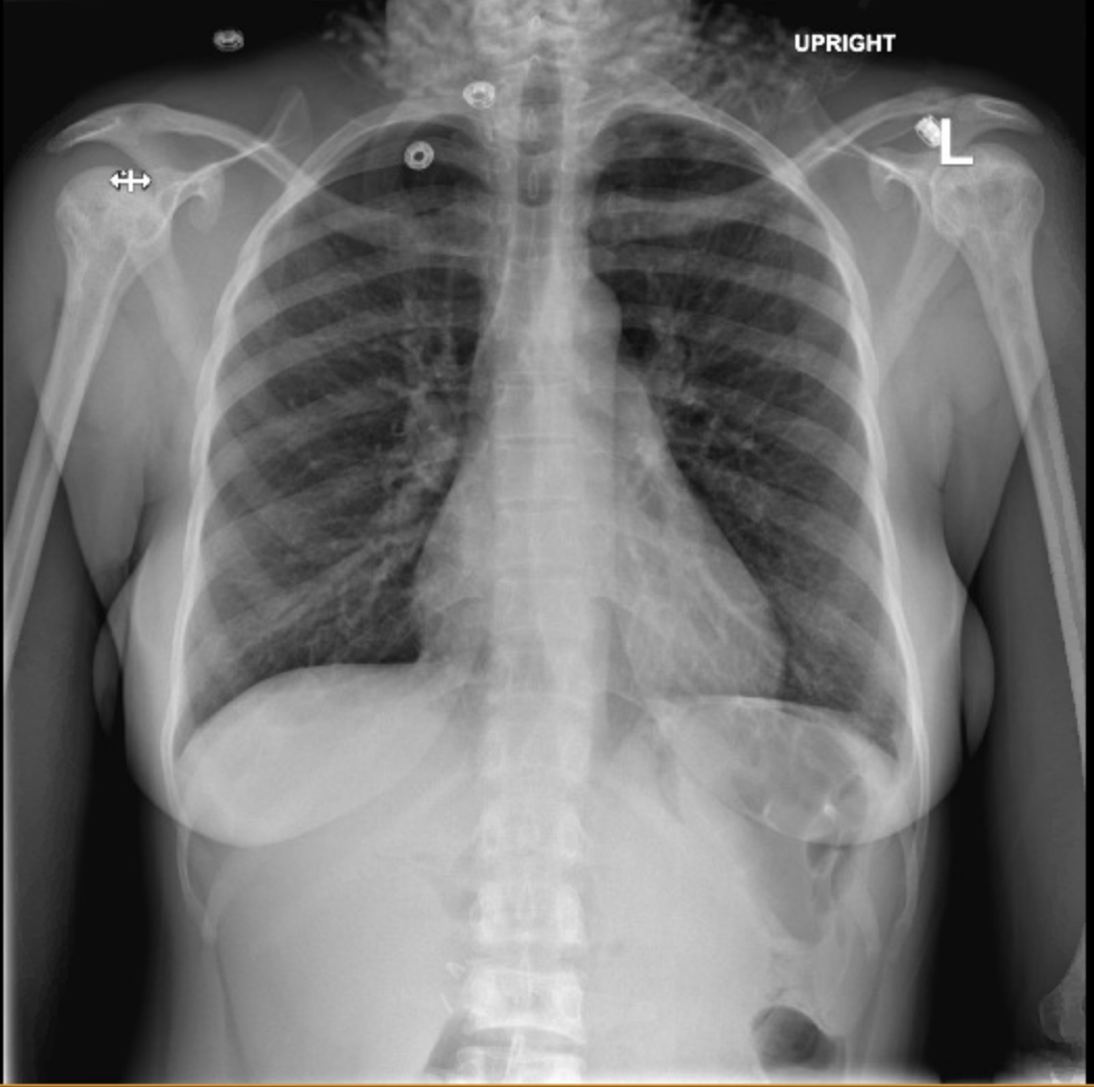
Patient’s chest X-ray on admission

Initial workup included electrocardiogram (EKG), troponin, basic blood work, a chest X-ray, and a CT scan of the chest, abdomen, and pelvis with contrast. EKG showed normal sinus rhythm with T-wave inversions in leads V2-V4. Troponin was negative. Hemoglobin was 6.4 g/dl (her baseline was around 8). Bilirubin was elevated to 10.7 mg/dl. Transaminitis was observed, aspartate aminotransferase (AST) - 214 IU/L, alanine aminotransferase (ALT) - 260 IU/L, alkaline phosphatase (ALP) - 129 IU/L. Chest X-ray did not show any evidence of consolidation, pleural effusion, or pneumothorax (Figure 1). CT chest with contrast was negative for pulmonary embolism (PE), and also negative for acute parenchymal lung disease. CT abdomen and pelvis with contrast was negative for acute intraabdominal pathology. The spleen was of normal size. She was transfused one unit of packed red blood cells (PRBC). Pain control was achieved with a morphine patient-controlled analgesia (PCA) pump.

Her hemoglobin dropped to 5.7 g/dl, so she was given two more units of PRBCs the following day. At that time, her oxygen requirements also increased to 2 liters via nasal cannula.

On the third day of her hospital course, her respiratory status severely declined requiring eventual intubation and transfer to the medical intensive care unit. Her vital signs at the time of transfer were as follows: blood pressure of 101/79, heart rate of 131, respiratory rate of 18 set by the ventilator, and oxygen saturation of 95%. She was afebrile.

**Figure 2:**
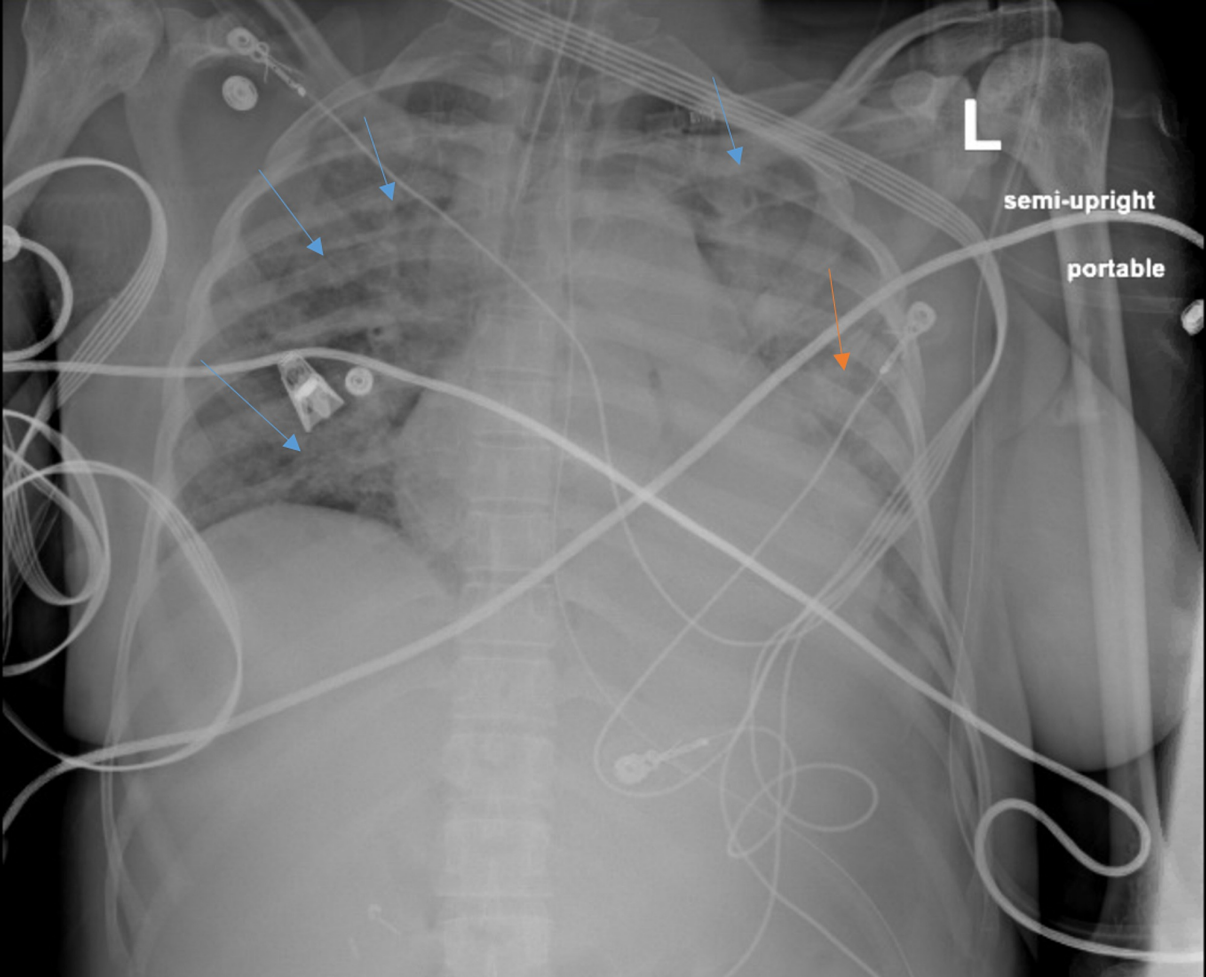
Patient’s chest X-ray after transfer to the ICU post-intubation. New diffuse bilateral infiltrates are noted (blue arrows), new consolidative changes in the left lower lobe are noted as well (orange arrow).

A repeat chest X-ray after transfer showed new diffuse bilateral infiltrates along with more consolidated changes in the left lower lobe that had developed since the previous exam (Figure 2). The patient was diagnosed with acute chest syndrome. Antibiotic treatment with Ceftriaxone and Azithromycin was initiated and Hematology was consulted who recommended PRBC transfusion to get her hemoglobin to 7 g/dl after which exchange transfusion was performed on two consecutive days. The percentage of hemoglobin SS was reduced to 8% after exchange transfusion.

The patient’s respiratory status also improved after the exchange transfusions and she was extubated the following day. As her respiratory status improved, her hemolysis markers did not correlate with her improving clinical status. Her reticulocyte count continued to trend downward and nucleated RBC count continued to trend upward after extubation along with an elevated LDH, persistently low haptoglobin, and worsening anemia. A peripheral smear was reviewed showing 1-2 schistocytes per High Powered Field. A diagnosis of hyperhemolysis syndrome was thus made and a decision was made to hold further transfusions and initiate the patient on methylprednisolone 40 mg twice daily.

Over the next four days, her reticulocyte count stabilized and nucleated RBC count decreased. LDH and bilirubin also decreased. Her hemoglobin stabilized to around 8 g/dl (Table 1). She was discharged with oral prednisone in stable condition. She was instructed to follow up with an outpatient hematologist to complete the steroid taper and her hemoglobin improved to her baseline of around 10 g/dl.

**Table 1:** Trend of lab values in our patient throughout her hospital course. Day 6 is highlighted because that is when methylprednisolone was initiated for suspected hyperhemolysis syndrome. LDH: Lactate dehydrogenase; nRBC: nucleated red blood cell; WBC: White blood cell.

Day	0	3	4	5	6	7	8	9	10	11
LDH (U/L)	696	>2,500	>2,500	>2,500	>2,500	>2,500	>2,500	1,909	1,991	1,745
Bilirubin (mg/dL)	10.7	11.0	4.4	-	3.5	3.3	2.0	1.1	1.3	1.3
nRBC per 100 WBC	2	21	50	81	61	38	32	31	23	19
Reticulocyte Count (m/mcL)	0.233	0.071	0.085	0.029	0.036	-	-	0.035	0.032	0.048
Hemoglobin (g/dL)	6.4	4.9	9.2	8.8	9.8	9.5	9.5	8.4	8.5	7.5
Haptoglobin (mg/dL)	<10	<10	-	-	<10	<10	<10	-	-	<10

## Discussion

Hyperhemolysis syndrome is a complication that affects patients with underlying hemoglobinopathies. Most documented cases involve patients with sickle cell disease [1]. However, other hemoglobinopathies such as thalassemia, hemoglobin C and hemoglobin SC disease can result in hyperhemolysis syndrome after blood transfusions [2]. Furthermore, hyperhemolysis syndrome can be seen in other hematologic disorders such as myelofibrosis, anemia of chronic disease, and lymphoma. Hyperhemolysis syndrome is diagnosed when severe anemia occurs paradoxically after a blood transfusion causing posttransfusion hemoglobin to be less than pretransfusion hemoglobin [3].

The clinical presentation to look for includes fever, jaundice, and severe pain [3]. Important lab values to look for include elevated lactate dehydrogenase, elevated bilirubin, and decreased reticulocyte count. Nucleated red blood cell count is also elevated [3]. Our patient exhibited all of these clinical features and lab values.

Hyperhemolysis syndrome can be broken down into two forms: the acute and delayed forms. This is based on the length of time from transfusion to the development of symptoms and formation of alloantibodies [3]. In the acute form, clinical symptoms are seen within seven days of the transfusion. Alloantibodies are usually not formed within this time frame, and thus a direct antiglobulin test to detect alloantibodies would likely be negative [3]. In the delayed form, clinical symptoms are seen beyond seven days after transfusion, and alloantibodies are more likely to have formed. A direct antiglobulin test in this case would likely be positive [3]. In our patient, the acute form was observed as symptoms were seen within seven days of transfusion, and an antibody test was negative.

Hyperhemolysis syndrome has been gaining prevalence as a diagnosis since 2009 when the UK Serious Hazards of Transfusion Scheme was published highlighting adverse reactions and events with transfusion of blood components in the UK [4]. The incidence of hyperhemolysis syndrome in sickle cell disease patients has not been clearly established to date [5]. Diamond et al. performed a case series of 18 patients with sickle cell disease who underwent partial exchange transfusion. Three of these 18 patients developed delayed hemolytic reactions consistent with hyperhemolysis syndrome [6]. There have also been studies to date elucidating an estimate for the prevalence of hyperhemolysis syndrome in pediatric patients with sickle cell disease. Talano et al. performed an 11-year retrospective chart review of patients with a discharge diagnosis of sickle cell disease who underwent a transfusion during their hospital course [7]. Each year, their center estimated transfusion to 162 pediatric patients with sickle cell disease. They identified a total of seven pediatric patients that suffered from hyperhemolysis syndrome over their 11-year retrospective study, a prevalence of about 0.39% [7]. A definitive prevalence for hyperhemolysis syndrome in sickle cell disease, both in adult and pediatric patients, has yet to be established.

A pathogenesis of hyperhemolysis syndrome has also not been established to date. Many different mechanisms have been proposed, with the most popular being “bystander hemolysis”. This phenomenon occurs when both native and donor red blood cells undergo hemolysis likely due to complement activation [3]. Other theories include an uptick in hemolysis by activated macrophages after transfusion, suppression of erythropoiesis, and increased expression of phosphatidylserine. When phosphatidylserine is expressed on the red blood cell surface, it results in increased likelihood of clearance of the red blood cell from circulation [3].

The current treatment for hyperhemolysis syndrome starts with holding transfusions to prevent further hemolysis. Corticosteroids and intravenous immune globulin (IVIG) can also help prevent further hemolysis and accentuate recovery of the patient’s hemoglobin levels [8]. Other additional treatment options reported include Rituximab [9], and Eculizumab, a C-5 convertase inhibitor, which inhibits complement activity thereby preventing complement-mediated RBC destruction [10].

## Conclusions

Hyperhemolysis syndrome can be a life-threatening complication of seemingly innocuous transfusion treatment. It is especially well documented in sickle cell disease, although it can be observed while transfusing patients with other hemoglobinopathies as well. The exact prevalence of hyperhemolysis syndrome in sickle cell disease has not been established and requires more investigation. With this case report, we aim to increase awareness of providers to this diagnosis and prevent critical anemia.
